# Unmet healthcare needs, access to services and experiences with health providers among persons with spinal cord injury in Australia

**DOI:** 10.1038/s41393-024-00997-4

**Published:** 2024-05-28

**Authors:** Samantha J. Borg, David N. Borg, Mohit Arora, James W. Middleton, Ruth Marshall, Andrew Nunn, Timothy Geraghty

**Affiliations:** 1 The Hopkins Centre, Menzies Health Institute Queensland, Griffith University, Brisbane, QLD Australia; 2 Division of Rehabilitation, Princess Alexandra Hospital, Brisbane, QLD Australia; 3 School of Exercise and Nutrition Sciences, Queensland University of Technology, Brisbane, QLD Australia; 4 John Walsh Centre for Rehabilitation Research, Northern Sydney Local Health District, St Leonards, NSW Australia; 5 The Kolling Institute, Faculty of Medicine and Health, The University of Sydney, Sydney, NSW Australia; 6 South Australian Spinal Cord Injury Service, Central Adelaide Local Health Network, Adelaide, SA Australia; 7 Faculty of Health and Medical Sciences, University of Adelaide, Adelaide, SA Australia; 8 Victorian Spinal Cord Service, Austin Health, Heidelberg, VIC Australia

**Keywords:** Rehabilitation, Risk factors

## Abstract

**Study design:**

Cross-sectional survey.

**Objectives:**

Appropriate and timely lifelong access to healthcare following a spinal cord injury (SCI) is critical, yet unmet healthcare needs in this population are common. Poor experiences with healthcare providers can be a barrier to health-seeking behaviour, and we hypothesised that there would be an association between unmet healthcare needs and care experiences. This study aimed to: (1) describe healthcare provider utilisation in the past year, unmet care needs and satisfaction with healthcare services; (2) explore the association between experiences with healthcare providers and unmet healthcare needs; and (3) explore the association between healthcare provider utilisation and participant characteristics, including unmet healthcare needs.

**Setting:**

Community.

**Methods:**

Analysis of data for 1579 Australians aged ≥ 18, who were ≥ 1-year post-SCI and living in the community. Bayesian penalised regression was used to model six binary outcomes: unmet healthcare needs; the use of general practitioners (GPs), allied health practitioners, rehabilitation specialists; medical specialists; and hospitalisations in the past 12-months.

**Results:**

Unmet needs were reported by 17% of participants, with service cost the common deterrent. There was evidence of an effect for provider experiences on unmet healthcare needs, but no evidence that unmet healthcare needs was associated with the use of GPs, allied health practitioners, and rehabilitation or medical specialists.

**Conclusions:**

Unmet healthcare needs were reported in the context of high healthcare use and large proportions of secondary conditions in a cohort with long-term SCI. Improved health access for people with SCI include better primary-secondary care collaboration is needed.

## Introduction

Lifelong access to healthcare services following a spinal cord injury (SCI) is critical for the prevention and management of secondary health complications that may otherwise reduce quality of life [1]. Common secondary conditions of SCI include high rates of depression, urinary tract infections, pressure injuries, pain and other chronic illnesses that typically occur with ageing [2–4]. These conditions require specialised management through comprehensive, multidisciplinary and primary health care arrangements [5]. While it is recognised that people with SCI generally require higher levels of healthcare than the general population, they also commonly report unmet healthcare needs. Access to care for people with SCI is highly variable [5], and barriers to care are common, including a lack of financial resources, and mobility and transportation difficulties [6–8].

Many mainstream and primary health care services do not have the capacity or means to manage people with SCI, due to their complex ongoing medical and rehabilitation needs. These factors further complicate the multi-faceted dynamic between health service use and unmet healthcare needs in people with SCI. Having services available in close proximity (i.e., potential access) and actually seeing the health practitioners (i.e., realised access) provide important perspectives regarding access, but are not comprehensive indicators of access [9]. A recent scoping review identified that measuring multiple dimensions of access is essential for a comprehensive understanding of healthcare access [10]. Other factors for measuring the breadth of health service access are also crucial, including the presence of unmet healthcare needs, which can directly result in poorer clinical or recovery outcomes [11]. A recent study identified unmet health needs as the most important factor to explain inequality in comorbidities experienced by people with SCI [12, 13]. Understanding a patients’ experiences with healthcare providers is also significant.

Poor prior health provider experiences are a well-documented barrier to health-seeking behaviour, engagement in care and adherence to treatment [12, 13]. In cases where health services are insufficient or of poor quality, people with SCI may need to rely on self-management and coordinating their own care [7]. As such, it is important to contextualise perceived and realised access to services, within the person’s experience of services. The relationship between unmet healthcare needs and satisfaction among persons with SCI has received limited scientific attention, and as such, was a focus of the current study.

The primary aims were to: (1) describe healthcare provider utilisation, unmet healthcare needs and satisfaction with healthcare services provided for an Australian cohort with SCI; and (2) explore the association between experiences with healthcare providers and unmet healthcare needs. The secondary aim of the study was to explore associations between healthcare provide utilisation and participant characteristics, including unmet healthcare needs. We hypothesised that people with SCI would have high levels of health service use but lower levels of satisfaction with services. We also hypothesised that those who had poorer healthcare experiences would be more likely to have unmet healthcare needs.

## Methods

The International Spinal Cord Injury (InSCI) community survey is a large-scale collaborative cross-sectional survey completed across 22 countries in 2017–18 [14, 15]. This study used data completed by Australian participants of the InSCI community survey (henceforth, Aus-InSCI) [16]. The full survey included 193 questions. A subset of these 193 questions related to use of healthcare practitioners, unmet needs, satisfaction with healthcare providers and details about participants’ primary health care provider. Further details of the Aus-InSCI survey are published elsewhere [16, 17].

### Study setting

This cross-sectional study was informed from 11 datasets of people with SCI supplied from nine data custodians. Data custodians from four Australian states participated: New South Wales, Queensland, South Australia and Victoria. Custodians included specialist SCI unit(s) or service(s) in each of the four participating states, three not-for-profit SCI consumer associations and a government insurance agency [16].

Databases were securely provided by custodians to an external institution (Curtin University) for linkage to identify and remove duplicates, creating a single master dataset [16]. This dataset was securely sent to the Australian Institute of Health and Welfare (AIHW) for linkage to the National Death Index. This was returned to Curtin University and following cleaning, de-duplication and removal of deceased individuals data, the individual re-identifiable datasets were returned to their respective data custodian for recruitment [16]. Full details on the secure data linkage process are described in the Aus-InSCI protocol paper [16].

Ethics was approved by the Northern Sydney Local Health District HREC (HREC/16/HAWKE/495) and AIHW Ethics Committee (EO2017/1/341). Implied consent was used for participants who completed surveys.

### Participants

Participants sourced from data custodian databases were eligible if they were aged 18 and over, living in the community, at least 12-months post SCI and were able to complete the questionnaire in English. Paper-based surveys were mailed to eligible participants in 2018 and could be completed as a hardcopy or online via enclosed unique participant login details. Follow-up reminders were mailed at 3- and 6-months after the initial contact for those participants who did not respond.

### Data measures

Survey questions on health service use and unmet healthcare needs were derived from the Model Disability Survey [18]. The specific questions are described below.

#### Healthcare provider utilisation

Service use was assessed with the question *‘who were the health care providers you visited in the community or hospital, or who visited you in your home, in the last 12-months?’*. Fifteen types of healthcare providers, including ‘other’, were listed. Participants also identified their main contact for SCI-specific problems (i.e., general practitioner, local specialist, spinal specialist, or other).

#### Experiences with healthcare providers

The survey collected information on participants’ last visit with a healthcare provider: “*For your last visit to a health care provider, how would you rate the following*: (1) *‘your experience being treated respectfully?*’, (2) *‘how clearly health care providers explained things to you?*’, and (3) *‘your experience being involved in making decisions for your treatment?’”*. Responses for these questions were: ‘very good’, ‘good’, ‘neither’, ‘bad’, or ‘very bad’.

#### Satisfaction with healthcare services provided

This was measured using three questions: *‘how satisfied are you with the services provided by (1) your general practitioners? (2) your local general hospital/s? (3) the Spinal Cord Injury Unit/Services in your state?’*. Participants provided a response on a 5-point Likert scale from ‘very satisfied’ to ‘very dissatisfied’.

#### Unmet healthcare needs

The following question was used as a proxy for unmet healthcare needs, *‘in the last 12 months, have you needed health care but did not get it?’*. Participants who responded ‘yes’ were classified as having unmet healthcare needs. Reasons for not being able to access healthcare were also collected (e.g., the cost of the service, unavailable services, inadequate provider skill).

#### Hospitalisations

Hospitalisations were collected based on the question: *‘over the last 12-months how many times were you a patient in a hospital, rehabilitation facility or care facility for at least one night?’*

General satisfaction was captured using the question: *‘in general, how satisfied are you with how the health care services are run in your area?’*. Participants provided a response on a 5-point Likert scale from ‘very satisfied’ to ‘very dissatisfied’.

#### Health measures

Fourteen secondary conditions were assessed, with responses given on a 5-point Likert scale ranging from 1 ‘no problem’ to 5 ‘extreme problem’. The 14 secondary conditions captured were: bowel, bladder and sexual dysfunctions; urinary tract infections; contractures; spasticity; pressure ulcers; respiratory problems; injury caused by loss of sensation; autonomic dysreflexia; postural hypotension; sleep and circulatory problems; and pain. Participants were given a score (between 0 and 14) based on the number of severe secondary conditions (that is, a response 4 or 5 ‘extreme problem’). A higher score on this scale indicates a greater number of severe secondary conditions.

#### Sociodemographic and injury characteristics

Sociodemographic factors included gender, age, marital status, education, rurality, daily household assistance being received (no/yes); weekly household income; and disability pension statues (no/yes) (see Table 1 for variable levels). Injury characteristics included injury level and completeness, injury aetiology (i.e., traumatic or non-traumatic) and time since injury (years).

**Table 1:** Variables included the six Bayesian penalised regression models.

Variable	Variable levels	Variables included in models					
		Unmet healthcare needs	GP use	Allied health use	Rehabilitation /SCI specialist use	Medical specialist use	Hospitalisations
Age	Continuous	Y	Y	Y	Y	Y	Y
Gender	Male, female	Y	Y	Y	Y	Y	Y
Geography	Capital city, rural area, remote area	Y	Y	Y	Y	Y	Y
Education	Primary/lower secondary, higher secondary, Certificate, Diploma, Bachelor, Masters/PhD	Y	Y	Y	Y	Y	Y
Marital status	single, married, divorced/widowed	Y	Y	Y	Y	Y	Y
Weekly household income	<$455; $456–$686; $687–$909; $910–$1,203; ≥ $1204	Y	Y	Y	Y	Y	Y
Daily professional household assistance	No, yes	Y	Y	Y	Y	Y	Y
Receiving disability pension	No, yes	Y	Y	Y	Y	Y	Y
Level & completeness	Incomplete paraplegia, complete paraplegia, incomplete tetraplegia, complete tetraplegia	Y	Y	Y	Y	Y	Y
Injury aetiology	Non-traumatic, traumatic	Y	Y	Y	Y	Y	Y
Time since injury	Continuous	Y	Y	Y	Y	Y	Y
No. severe secondary conditions	Continuous	Y	Y	Y	Y	Y	Y
modified-SCIM-SR	Continuous	Y	Y	Y	Y	Y	Y
Unmet healthcare needs	No, yes	–	Y	Y	Y	Y	Y
Provider experience….							
Being treated respectfully	Very good, good, neither, bad	Y	–	–	–	–	–
Clarity of explanations from health providers	Very good, good, neither, bad	Y	–	–	–	–	–
Being involved in treatment decisions	Very good, good, neither, bad	Y	–	–	–	–	–

*GP* general practitioner, *modified-SCIM-SR* modified Spinal Cord Independence Measure (SCIM), self-reported, *Y* yes.

#### Functional independence

The Spinal Cord Independence Measure (SCIM) is a 16-item measure assessing mobility, self-care, and respiration. A score is calculated ranging from 0 to 100 where higher scores indicate higher functional independence [19]. The InSCI survey used a modified, self-reported version of the SCIM (henceforth, modified-SCIM-SR). A subset of the SCIM with scores ranging from 0 to 66 was used. This included all self-care (feeding, bathing, grooming), bowel and bladder management, and some mobility measures (bed to wheelchair transfers, moving moderate distances, activities able to be performed without assistance or aids). This version was rescaled to the original measure (i.e., 0–100) for easy interpretation, where higher scores again indicate greater functional independence [19].

### Data analysis

All analyses were undertaken in R (version 4.1.1) [20] using the RStudio environment (version 1.4.1717) [21]. Sociodemographic and injury characteristics, income and education, measures of health, health service use, unmet healthcare needs and satisfaction with healthcare services provided with services were descriptively summarised as count and percent or median and first and third quartile.

Bayesian penalised regression was used to model the six binary outcome variables. Penalised regression guards against overfitting [22], with the aim to shrink small non-important effects to zero while maintaining true, larger effects. Shrinkage (horseshoe) prior distributions with three degrees of freedom were specified for regression coefficients in all models. A logit link function was used to connect the Bernoulli distribution with the regression coefficients. Bayesian models were fit with Stan [23] using the *brms* interface [24].

The six outcomes were: (1) unmet healthcare needs; (2) general practitioner use; (3) allied health practitioner use; (4) medical specialist use; (5) rehabilitation specialist use (inclusive of SCI specialists); and (6) hospitalisations in the past 12-months. Five of the six models included the same 14 predictor variables: age, gender, geographical area, education, daily professional household assistance, receives disability pension, marital status, weekly household income, level and completeness, injury aetiology, time since injury, number of severe secondary conditions, modified-SCIM-SR and unmet health care needs (Table 1). The model for unmet healthcare needs also included provider experiences (Table 1).

There were missing values in 14 of the 16 variables included in models, with the percentage missing ranging from 0.1% to 19.5% (Suppl. 1) [25]. Values were assumed to be missing at random. Missing values were imputed using predicted mean matching, with the results averaged over five imputed datasets.

Posterior estimates were generated using Markov chain Monte Carlo procedures (50,000 iterations, 4 chains and thinned by a factor of 5) and are reported as the mean and 95% credible interval (CrI). Parameter estimates are shown on the logit scale in figures and are reported as odds ratios in supplementary tables, along with the posterior probability that the odds ratio was greater than the null value of 1 (Pr OR > 1) and less than the null value of 1 (Pr OR < 1). The results were interpreted using estimation methods [26].

## Results

Sample characteristics are reported in Table 2 for 1579 responders. Participants were generally male, had a median age of 59 years, and were mostly married (59%). The median time since injury was 13 years and most participants had incomplete paraplegia (37%).

**Table 2:** Participant demographic, injury, living arrangements and economic characteristics.

Characteristic	Total (*N* = 1579)	Unmet healthcare needs (*N* = 1514)	
		No unmet needs, *n* = 1260	Unmet needs, *n* = 254
***Demographic characteristics***			
Male, *n* (%)	1157 (73)	937 (74)	171 (67)
Age, median (IQR)	59 (48–68)	60 (49–69)	53 (44–63)
Marital status, *n* (%)			
Single	386 (25)	282 (22)	82 (32)
Married/partnership	931 (59)	782 (62)	124 (49)
Divorced/widowed	257 (16)	195 (15)	48 (19)
***Injury characteristics***			
Time since injury, median (IQR)	13 (6–25)	13 (6–25)	12 (6–24)
Lesion characteristics, *n* (%)			
Paraplegia, incomplete	542 (37)	436 (37)	96 (40)
Paraplegia, complete	362 (24)	301 (25)	49 (20)
Tetraplegia, incomplete	449 (30)	347 (29)	78 (32)
Tetraplegia, complete	128 (9)	104 (9)	19 (8)
Cause of injury, *n* (%)			
Traumatic	1,306 (84)	1,050 (84)	209 (83)
Non-traumatic	257 (16)	204 (16)	44 (17)
*Living situation*			
Rurality, *n* (%)			
Capital city	864 (57)	725 (58)	132 (53)
Rural area	390 (26)	316 (25)	71 (29)
Remote area	256 (17)	204 (16)	45 (18)
***Socioeconomic factors***			
Weekly household income, *n* (%)			
Less than $455	356 (26)	265 (24)	74 (32)
$456–$686	358 (26)	288 (26)	58 (25)
$687–$909	292 (21)	234 (21)	50 (22)
$910–$1,203	258 (19)	215 (20)	34 (15)
$1204 and above	112 (8)	97 (9)	13 (6)
Receiving disability pension	722 (47)	566 (45)	140 (56)
Education,^a^ *n* (%)			
Primary or lower secondary	495 (32)	414 (33)	58 (23)
Higher secondary	207 (13)	166 (13)	34 (14)
Certificate	292 (19)	226 (18)	57 (23)
Diploma	179 (12)	137 (11)	34 (14)
Bachelor or equivalent	239 (15)	188 (15)	42 (17)
Masters or PhD	137 (9)	109 (9)	22 (9)

*IQR* interquartile range.

*Note*. There are 65 (4%) missing cases in the ability to get care—numbers will not equate to that in total column.

^a^Includes vocational education and training; certificates; and tertiary preparation.

Healthcare provider utilisation, hospitalisations and unmet healthcare needs in the past 12-months are summarised in Table 3. Less than half (45%) of the sample had 1 or more hospitalisations in the past 12 months. Unmet needs were reported by 17% of participants. For these individuals, service costs (30%, *n* = 77), inadequate provider skills (19%) and service unavailability were the most common deterrents to accessing services (19%) (see Table 3). General practitioners were the most frequently accessed healthcare provider (80%), followed by pharmacists (54%) and physiotherapists (43%).

**Table 3:** Unmet healthcare needs, healthcare providers accessed and hospitalisations in the past 12-months.

Variable	Total (*N* = 1579)
	*n* (%)
**Healthcare providers seen in the past 12-months (*n*** = **1579)**	
General practitioner	1213 (80)
Pharmacist	815 (54)
Physiotherapist	655 (43)
Other specialist physician (e.g., surgeon)	600 (39)
Dentist	586 (39)
Occupational therapist	570 (37)
Rehabilitation specialist	522 (34)
Home healthcare worker	483 (32)
Nurse	491 (32)
Psychologist	182 (12)
Chiropractor or alternative medicine practitioner	115 (8)
Dietitian	158 (10)
Social worker	144 (9)
Other	153 (10)
No providers	72 (5)
**Number of hospitalisations in the past 12-months**^**a**^	
No hospitalisations	787 (55)
1-2 hospitalisations	489 (34)
3 or more hospitalisations	168 (12)
**Unmet healthcare needs in the past 12-months**^**b**^ **(*n*** = **254)**	
Could not afford the cost of visit	77 (30)
Service unavailability	48 (19)
Inadequate provider skill	49 (19)
Thought they were not sick enough	45 (18)
Inadequate provider drugs or equipment	37 (15)
Other reasons	35 (14)
Previous badly treated	34 (13)
Did not know where to go	30 (12)
Tried but were denied healthcare	31 (12)
Transport unavailability	22 (9)
Could not take time off work or other commitments	21 (8)
Could not afford transport	13 (5)

*Note*. Percentages are based on complete responses and may not equate to the percentage among the total sample.

^a^Number of times in a hospital, rehabilitation facility or other care facility for at least one night.

^b^Participants could select all relevant reasons relating to their unmet needs.

Satisfaction with healthcare and experiences are summarised in Table 4. General practitioners were the main point of contact for SCI-specific problems (58%), followed by specialist spinal physicians (31%). While most participants (81%) reported being satisfied or very satisfied with their general practitioner, only 67% and 62% respectively expressed the same level of satisfaction with statewide SCI services and local general hospital services.

**Table 4:** Main health service contact, and satisfaction with healthcare and experiences.

Variable	*N* = 1579
**Main contact for SCI-specific problems,** ***n*** **(%)**	
General practitioner	865 (58)
Local specialist^a^	116 (8)
Spinal specialist^b^	464 (31)
Other	52 (3)
**Satisfaction with general practitioner,** ***n*** **(%)**	
Satisfied or very satisfied	1229 (81)
Neither	219 (14)
Dissatisfied or very dissatisfied	63 (4)
Do not use this service	8 (1)
**Satisfaction with state-wide SCI services,** ***n*** **(%)**	
Satisfied or very satisfied	1021 (67)
Neither	223 (15)
Dissatisfied or very dissatisfied	149 (10)
Do not use this service	127 (8)
**Satisfaction with local general hospital services,** ***n*** **(%)**	
Satisfied or very satisfied	936 (62)
Neither	244 (16)
Dissatisfied or very dissatisfied	116 (8)
Do not use this service	216 (14)
**Experience being treated respectfully,** ***n*** **(%)**	
Satisfied or very satisfied	1321 (90)
Neither	93 (6)
Dissatisfied or very dissatisfied	55 (4)
**Clarity of explanations by healthcare provider,** ***n*** **(%)**	
Satisfied or very satisfied	1283 (88)
Neither	130 (9)
Dissatisfied or very dissatisfied	53 (4)
**Experience of being involved with treatment decisions,** ***n*** **(%)**	
Satisfied or very satisfied	1251 (85)
Neither	156 (11)
Dissatisfied or very dissatisfied	59 (4)

*SCI* spinal cord injury.

^a^Including rehabilitation specialists, urologists, neurologists.

^b^Working in a specialist SCI service or unit.

### Modelling outcomes

#### Unmet healthcare needs

**Fig. 1:**
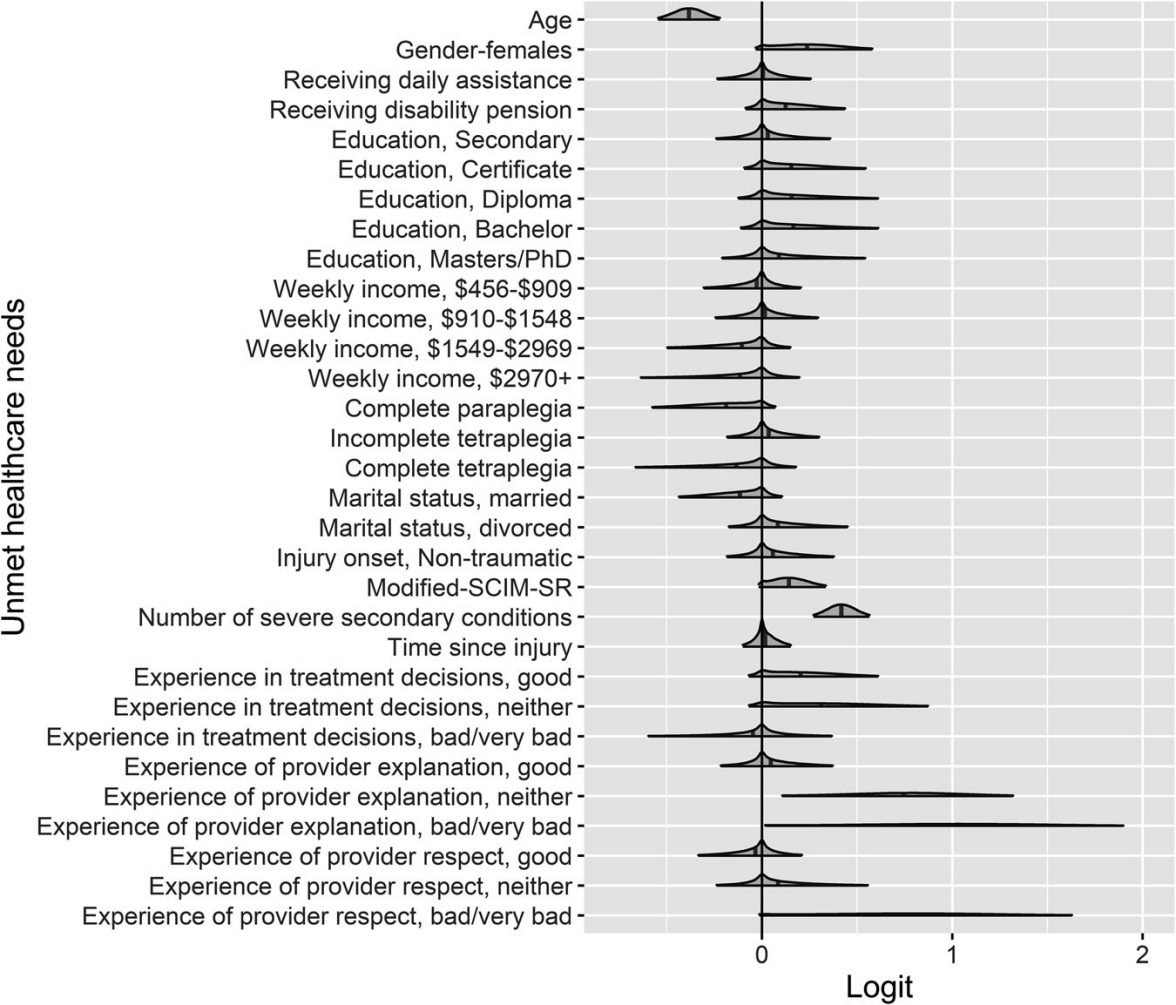
Parameter estimates from the Bayesian model for Unmet healthcare needs. Figure note: The grey shaded area is the smoothed density distribution of the data, where height is proportional to the number of data points that fall within that part of the distribution. Distributions are cut at the 95% credible interval. Modified-SCIM-SR = modified Spinal Cord Independence Measure (SCIM), self-reported.

**Fig. 2:**
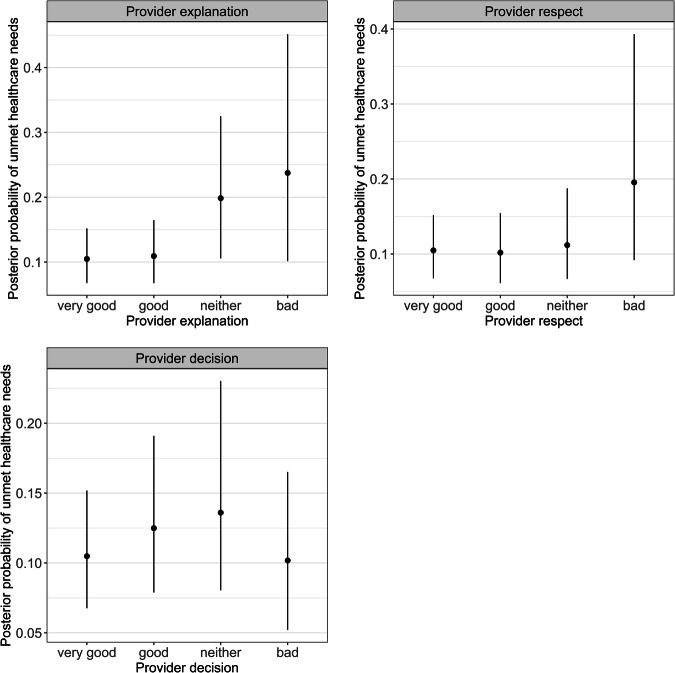
The marginal effects of the unmet healthcare needs. Figure note: The posterior mean (circle) and 95% credible interval (error bar) probability of unmet needs, according to (1) how clearly healthcare providers explained things to individuals (top left); (2) individual experience of being treated respectfully (top right); and (3) experience of being involved in making decisions regarding treatment(s) (bottom).

Being female, having greater functional capacity (i.e., higher modified-SCIM-SR scores) and a higher number of severe secondary conditions were associated with unmet healthcare needs (Fig. 1 and Supplementary Fig. 2). Older individuals and those with complete paraplegia were less likely to report unmet healthcare needs (Fig. 1 and Supplementary Fig. 2). Some instances of experiences with providers were also related to unmet needs (as a primary outcome of the study, marginal effects for treatment experiences are visualised in Fig. 2). Specifically, individuals with good or neutral experiences with being involved in their treatment decisions were more likely to report unmet healthcare needs (Fig. 2 and Supplementary Fig. 2). Those who reported that the clarity of explanations from health providers as *bad* or *neutral* were also more likely to report unmet healthcare needs (Fig. 2 and Supplementary Fig. 2). Finally, those who identified bad experiences of being treated respectfully had 2.1 times higher odds of reporting unmet healthcare needs (Fig. 2 and Supplementary Fig. 2).

#### General practitioner use

**Fig. 3:**
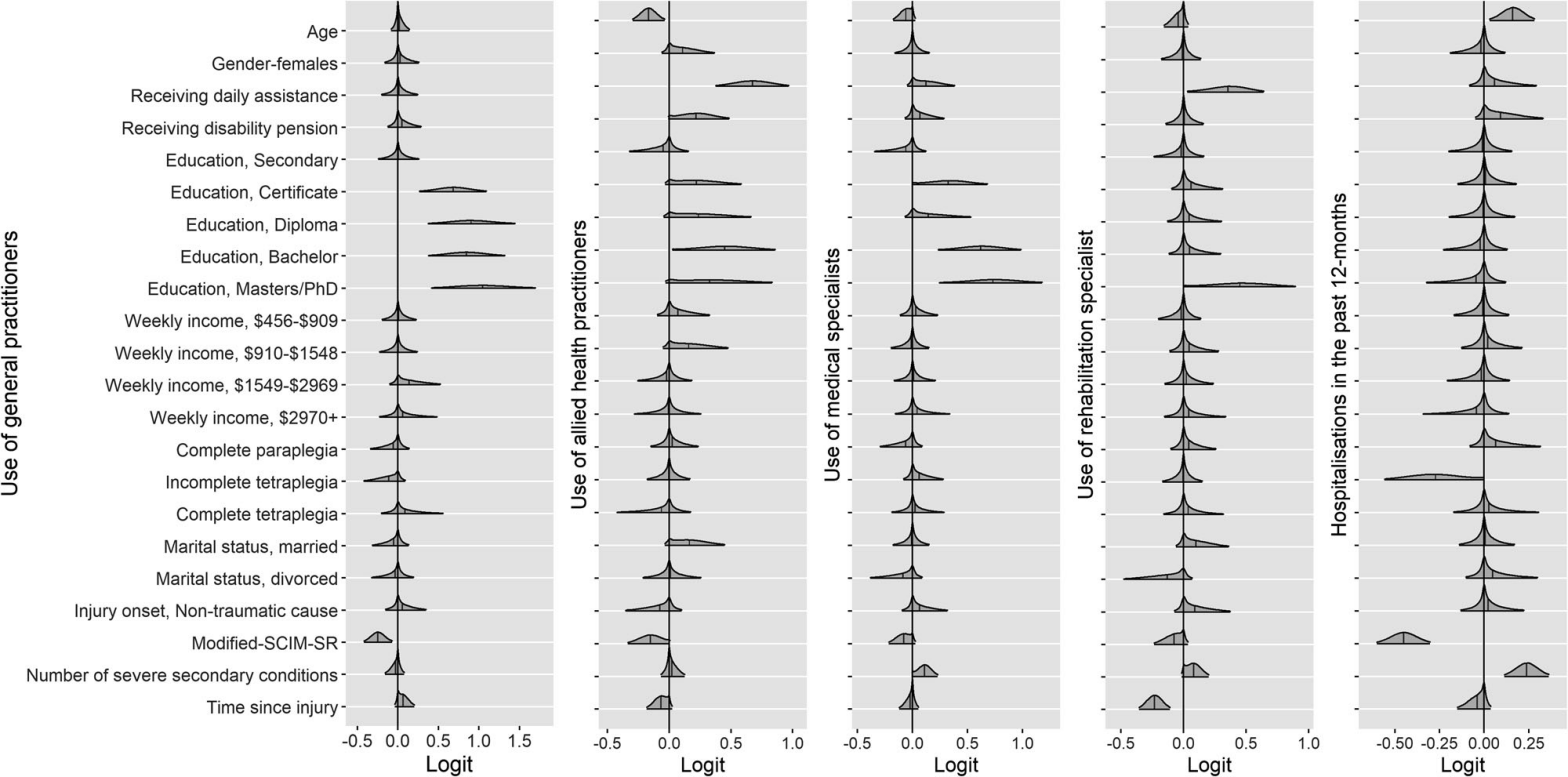
Parameter estimates from the Bayesian models for: General practitioners, Allied health practitioners, Medical specialists, Rehabilitation and/or SCI specialists and Hospitalisations in the past 12-months. Figure note: In each distribution, the vertical line indicates the posterior mean. The grey shaded area is the smoothed density distribution of the data, where height is proportional to the number of data points that fall within that part of the distribution. Distributions are cut at the 95% credible interval. Modified-SCIM = modified Spinal Cord Independence Measure.

A longer time since injury and higher education levels (Certificate, Diploma, Bachelor, Masters or PhD) were associated with greater odds of having used a general practitioner (Fig. 3 and Supplementary Fig. 3). Those with poorer functional status (i.e., modified-SCIM-SR) were less likely to have used a general practitioner (Fig. 3 and Supplementary Fig. 3).

#### Allied health use

Receiving daily professional household assistance, receiving a disability pension, being married, having higher education levels (i.e., Certificate, Diploma, Bachelor, Master’s or PhD) and living rurally were associated with greater odds of having seen an allied health practitioner in the previous 12-months (Fig. 3 and Supplementary Fig. 4). Older individuals, those with a greater functional capacity (i.e., modified-SCIM-SR) and a longer time since injury were less likely to have utilised allied health practitioners in the past 12-months (Fig. 3 & and Supplementary Fig. 4).

#### Rehabilitation specialist use

Individuals with a Master’s or PhD degree, those receiving daily household assistance and those with a higher number of severe secondary conditions were more likely to have visited a rehabilitation specialist (Fig. 3 and Supplementary Fig. 5). Those with a longer duration of SCI, greater functional status (i.e., higher modified-SCIM-SR score), and living in a rural or remote area were less likely to have seen a rehabilitation specialist in the past 12-months (Fig. 3 and Supplementary Fig. 5).

#### Medical specialist use

Those with a higher education level (i.e., Certificate, Bachelor, Master’s or PhD), receiving daily household assistance and a higher number of severe secondary conditions were more likely to have seen a medical specialist (Fig. 3 and Supplementary Fig. 6). Older people and those with a greater functional capacity (i.e., higher modified-SCIM-SR score) were less likely to have seen a medical specialist (Fig. 3 and Supplementary Fig. 6).

#### Health provider use and unmet healthcare needs

We did not find any evidence of an effect between the use of general practitioners, allied health practitioners, and rehabilitation or medical specialists for unmet healthcare needs (Fig. 3). The posterior probability that the regression coefficient for unmet healthcare needs was greater than 0 (i.e., no effect) in these models was 0.42, 0.31, 0.62, 0.73, 0.49, respectively (Supplementary 3–7).

#### Hospitalisations in the past 12-months

Older individuals and those with a higher number of severe secondary conditions were more likely to be hospitalised in the past 12-months (Fig. 3 and Supplementary Fig. 7). Those with a greater functional status (i.e., higher modified-SCIM-SR score) and incomplete tetraplegia were less likely to have been hospitalised (Fig. 3 and Supplementary Fig. 7).

## Discussion

This study focused on health service use, unmet healthcare needs and satisfaction with healthcare services provided for Australians living with SCI in the community. Unmet needs were reported among 1 in 5 participants. Moderate to high levels of satisfaction were reported by participants for GPs, state-wide SCI services and local hospitals. We found that experiences with healthcare providers were related to unmet healthcare needs.

On the surface, there appeared to be relatively high levels of realised access (Table 3). This included levels of access to GPs (80%) comparable to other SCI cohorts in the United States (US) (84%) [27] and Switzerland (82%) [28]. However, a recent Australian study identified that more than half of people with SCI would use a different GP if they were more knowledgeable (compared to their current GP) about their condition [29], suggesting areas for improvement regarding utilisation of GPs. Access to specialist services were less comparable to previous international findings with a third of participants reporting accessing SCI/rehabilitation specialists, compared to 41% and 54% respectively in Swiss [28] US cohorts [27]. Utilisation of other types of specialists was also lower, 39% compared to 51% in the US, as was [27] utilisation of allied health, dental and psychology services. Thirty-nine percent of study participants reported accessing dental services in the previous 12-months (Table 3). This was considerably lower than rates reported among a Swiss SCI cohort (76%) [30] and a traumatic SCI cohort in the US (57%) [27]. Relatively few participants accessed psychology (12%) and dietetic (10%) services in the previous year. This is worrying given the elevated rates of mental health problems [31, 32] and concerns with undernutrition and obesity [33] in people with SCI. Findings from this study also suggest that although levels of health service use collectively appear relatively high, there may be lower utilisation of important health provider services among older people or those with longer-term SCI. Older people with SCI were also more likely to have been hospitalised in the previous 12-months.

Unmet healthcare was reported by 17% of responders (Table 3). The broader InSCI survey project was conducted across 22 countries, allowing for cross-country comparison of reported rates of unmet healthcare needs among persons with SCI. Of the 22 countries that participated, Australia had the 8th highest rate of unmet healthcare needs, while the lowest levels of unmet needs were reported among Spanish participants (7%) [34]. Although the rate in Australia is much below that of Morocco (62%), South Korea (28%) or South Africa (27%), it remains a cause for concern that unmet needs were present in approximately 17% of participants. Further, as is acknowledged in the cross-country comparison, we similarly recognise that the use of self-reporting could result in bias and potential under-reporting of this issue [34]. Responder and non-responder characteristics were previously compared for Australian responders. There was higher participation from people in larger, more populated regional areas [16], which may further suggest under-representation from those in more isolated rural areas, where service access is known to be limited.

The potential for service under-utilisation cannot be appropriately considered without being contextualised to the reported reasons for unmet needs. The most common reasons identified for healthcare needs being unmet were due to service costs (30%), inadequate provider skills (19%) and service unavailability (19%). While the exact nature and extent of service availability for people with SCI in and across regions in Australia was not known, the presence of service unavailability within this population is not surprising. A study in the South-East Queensland region of Australia identified highly variable access to health services for people with acquired disability (inclusive of participants with SCI), where some of the poorest potential access was seen in relation to psychology services [5]. The study further identified various ‘hotspots’ of inequality resulting from areas of low healthcare access coinciding with areas with high prevalence of people with disability [5]. The associated inadequacy of resources, relating to reduced availability of both health services and transportation (identified by 9% of study participants), was considered to likely also be a priority in other areas in Australia, and requires policy change at a system level.

We found no evidence that use of health providers was associated with unmet healthcare needs, however, there was evidence of an effect for a range of participant characteristics on unmet needs. Females, those with a higher functional status and those with a greater number of secondary conditions was associated with higher odds of having unmet healthcare needs (OR = 1.3; Fig. 1 and Supplementary Fig. 2). A 2019 Swiss study of community-based persons with SCI supports this finding of higher unmet needs among females (at an incidence rate ratio of 1.3 compared to males) [35], pointing to greater support for females with SCI to better meet their health care needs. Unmet healthcare needs in those with higher physical functioning has also been previously reported. In particular, people with incomplete paraplegia who had lower satisfaction with fulfilment of healthcare needs and preferred to seek treatment at specialist SCI centres [8]. This may, at least in part, be due to reduced knowledge and awareness among non-SCI specialist healthcare providers about the less visible health care needs of people with high physical functioning. For example, people with cauda equina syndrome who may be ambulant but have significant hidden disability related to bladder, bowel and sexual dysfunction [36]. The unmet needs seen among those with a greater number of secondary conditions suggests people have difficulty dealing with a disproportionately high burden of health problems. More generally, the higher unmet needs in this cohort in conjunction with an underutilisation of crucial health services emphasise a need for focusing on long-term general health strategies and better access to both primary care and specialist SCI services as a means for preventing secondary conditions and protecting against unmet healthcare need.

A previous study in this same SCI cohort identified a high prevalence of secondary conditions. Frequent conditions were pain (85%), sexual dysfunction (79%) and sleep and bowel problems (78% each) [17]. Some highly prevalent secondary conditions, including sexual dysfunction and sleep problems may not be recognised as priorities by primary care clinicians to the same extent as other secondary conditions, such as pressure injuries and pain. However, given that people with SCI are predominantly managed in the community, where GPs are often the main provider or primary point of contact [4, 37, 38], ensuring an adequate mix of knowledge, resources, time and fit between primary care and specialist SCI services to both prevent and manage all relevant SCI-related problems is critical. People with SCI have previously identified that difficulties with accessing GPs has led to emergency departments attendance when GPs were not available [39]. Given that people with SCI often describe their healthcare access journey as an ‘uphill battle’ [39], a model of integrated care needs to be implemented across the health system. This is particularly critical when dealing with a group that experience a high proportion of secondary health conditions that can quickly deteriorate.

Satisfaction with care is often an underreported aspect of healthcare use. Although dissatisfaction with services was relatively low in the current study, greater levels of dissatisfaction were expressed in relation to state-wide SCI services and local hospital services over that of GPs (Table 4). Specialist SCI services are well established across Australian states, however, in view of the findings from this study indicating a potential supply-demand mismatch, a review of current levels of resourcing and service provision is warranted. This is particularly relevant with respect to outpatient, outreach and telehealth services.

This study identified several important findings that have implications for health services for people with SCIs in Australia: (1) there were relatively high levels of unmet healthcare needs, prominent among those with a higher number of secondary conditions; (2) there was evidence of a cross-sectional association between satisfaction with healthcare provider and unmet needs, including higher unmet healthcare needs for individuals who were not treated respectfully by healthcare providers; (3) higher dissatisfaction expressed towards statewide SCI units/services (compared to satisfaction with GPs); (4) potential under-utilisation of healthcare providers in the context of higher service needs among a population with long-term SCI; and (5) multi-faceted reasons for unmet healthcare needs, including inadequate provider skills or equipment and poor treatment experiences with health professionals. In the context of these key findings, we have provided recommendations, which are further supported by the issues identified in the broader literature among SCI populations relating to service access; and in line with person-centred approaches to healthcare.

### Recommendations

A main recommendation must be to improve shared care collaboration to enhance effective communication, interdisciplinary collaboration, and timely access to necessary services and is a responsibility shared between GPs and specialist SCI clinicians. Recent international literature supports this need for greater collaboration between primary, secondary, and tertiary healthcare systems for people with SCI, with the aim of being able to expand on pre-existing healthcare infrastructure [40, 41]. Additional recommendations for better care include:

1. adopting a person-centred approach, where people with SCI are actively involved in their care planning and decision-making;
2. offering specialised training and upskilling for GPs on SCI management [42]; and
3. delivering the right service at the right time, in the right place and by the right provider, thus developing more personalised rehabilitation pathways to allow for tailored interventions and therapies that address the goals of each individual with SCI, leading to improve healthcare access and health outcomes.

### Study limitations

The cross-sectional study design makes it difficult to interpret the direction of some associations in a cause-effect manner. Further, the survey did not capture an individuals’ funding information. Funding sources, for example, the national injury insurance schemes or broader disability funding schemes, including the National Disability Insurance Scheme, are complex but can include subsidisation of certain health providers, especially for allied health [43]. Funding though these types of schemes may directly impact an individuals’ capacity to engage with various health services.

## Conclusions

This study showed relatively high levels of unmet healthcare needs in the context of high healthcare use, and large proportions of secondary conditions in long-term SCI. This indicates an urgent need for streamlining and improving health systems and services to increase healthcare accessibility and meet the needs and expectations of people with SCI. Investigation into specific healthcare reform may be needed to better support any system changes. Nonetheless, initial recommendations include greater shared GP and SCI specialist responsibility and system-focused approaches to improve health service resourcing, availability and access.

### Supplementary information

Supplementary materials

## Data Availability

De-identified data is available upon request and with permission gained from the Aus-InSCI Community Survey National Scientific Committee.
