# Supplementary material for: Unmet healthcare needs, access to services and experiences with health providers among persons with spinal cord injury in Australia
Source: Spinal Cord. 2024 May 28;62(7):396–405. doi: 10.1038/s41393-024-00997-4 (PMC11230905; doi:10.1038/s41393-024-00997-4)
Supplement: Supplementary file 1 — Supplementary materials [file 41393_2024_997_MOESM1_ESM.docx]

**Supplemental 1.** Missing data


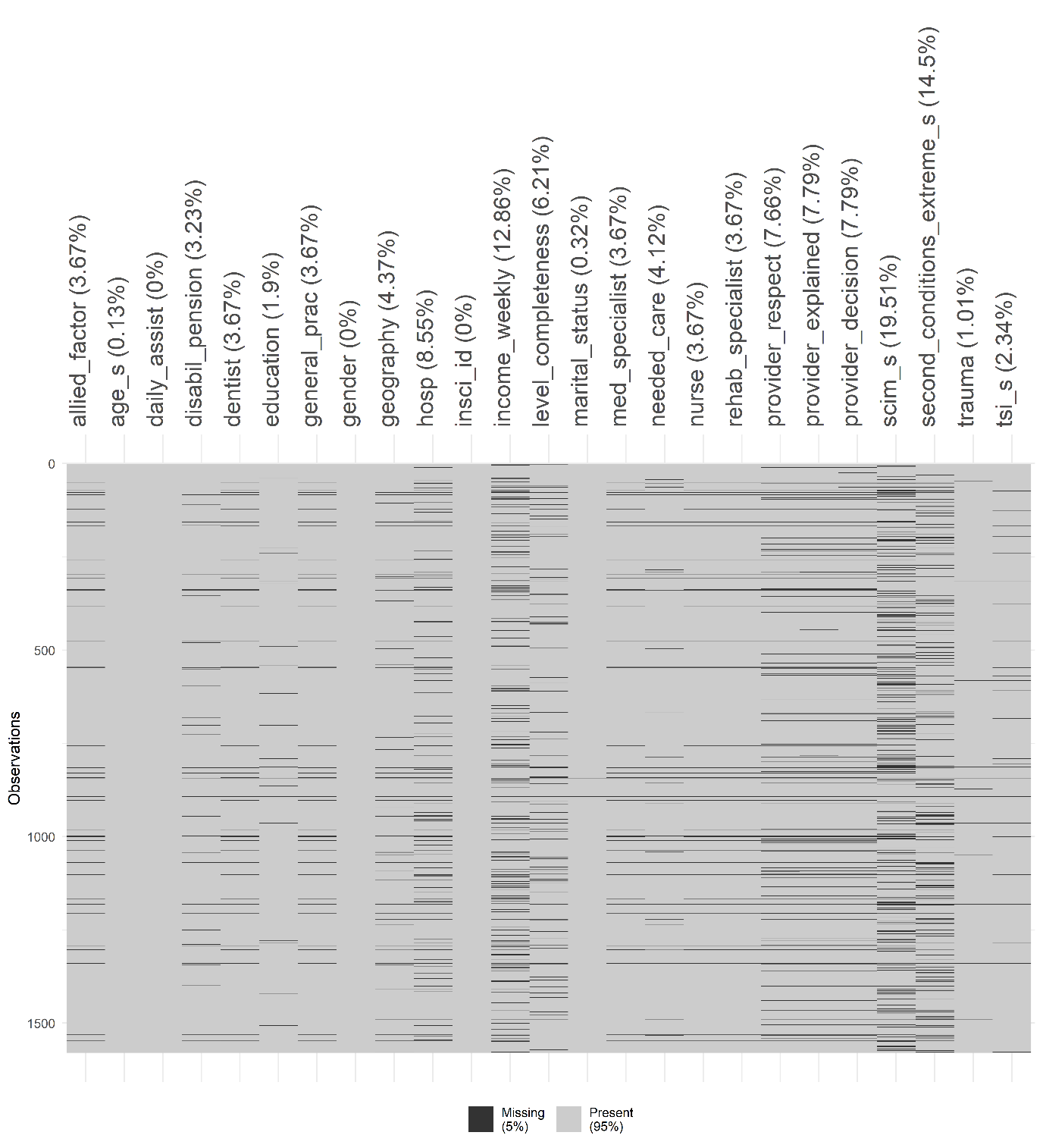


**Supplemental 2.** Unmet healthcare needs model outputs

| Variable | OR | 95% CI | | Pr OR <1 | Pr OR >1 |
| --- | --- | --- | --- | --- | --- |
|  |  | **Lower** | **Upper** |  |  |
| Age | **0.68** | **0.58** | **0.80** | **1.00** | **.00** |
| Females (ref is males) | **1.27** | **0.97** | **1.79** | **.07** | **.93** |
| Receiving daily assistance | 1.01 | 0.79 | 1.29 | .48 | .52 |
| Receiving disability pension | 1.13 | 0.92 | 1.55 | .18 | .82 |
| Education, |  |  |  |  |  |
| Primary/lower secondary (ref) | 1.0 |  |  |  |  |
| Secondary | 1.03 | 0.79 | 1.43 | .42 | .58 |
| Certificate | 1.17 | 0.91 | 1.73 | .17 | .83 |
| Diploma | 1.17 | 0.88 | 1.85 | .20 | .80 |
| Bachelor | 1.18 | 0.89 | 1.84 | .19 | .81 |
| Masters/PhD | 1.10 | 0.81 | 1.73 | .32 | .68 |
| Geography, |  |  |  |  |  |
| Capital city/metropolitan area (ref) | 1.0 |  |  |  |  |
| Rural area | 1.06 | 0.86 | 1.40 | .32 | .68 |
| Remote areas | 1.06 | 0.84 | 1.45 | .35 | .65 |
| Weekly household income, |  |  |  |  |  |
| <$455 (ref) | 1.0 |  |  |  |  |
| $456 to $909 | 0.97 | 0.74 | 1.23 | .58 | .42 |
| $910 to $1,548 | 1.02 | 0.78 | 1.34 | .45 | .55 |
| $1,549 to $2,969 | 0.90 | 0.60 | 1.16 | .73 | .27 |
| >$2,970 | 0.89 | 0.53 | 1.22 | .70 | .30 |
| Level and completeness, |  |  |  |  |  |
| Incomplete paraplegia (ref) | 1.0 |  |  |  |  |
| Complete paraplegia | **0.83** | **0.56** | **1.07** | **.87** | **.13** |
| Incomplete tetraplegia | 1.04 | 0.83 | 1.36 | .39 | .61 |
| Complete tetraplegia | 0.88 | 0.51 | 1.20 | .73 | .27 |
| Marital status, |  |  |  |  |  |
| Single (ref) | 1.0 |  |  |  |  |
| Married or cohabitating | 0.89 | 0.65 | 1.11 | .80 | .20 |
| Divorced or widowed | 1.09 | 0.84 | 1.56 | .30 | .70 |
| Modified-SCIM-SR | **1.15** | **0.98** | **1.40** | **.06** | **.94** |
| Number of severe secondary conditions | **1.52** | **1.31** | **1.76** | **.00** | **1.00** |
| Injury onset, |  |  |  |  |  |
| Traumatic (ref) | 1.0 |  |  |  |  |
| Non-traumatic | 1.06 | 0.83 | 1.46 | .35 | .65 |
| Time since injury | 1.02 | 0.90 | 1.16 | .39 | .61 |
| Experience of being involved in treatment decision making during last healthcare provider visit, |  |  |  |  |  |
| Very good (ref) | 1.0 |  |  |  |  |
| Good | **1.23** | **0.94** | **1.84** | **.12** | **.88** |
| Neither | **1.37** | **0.94** | **2.40** | **.10** | **.90** |
| Bad/very bad | 0.96 | 0.55 | 1.45 | .56 | .44 |
| Clarity of explanation during last healthcare provider visit, |  |  |  |  |  |
| Very good (ref) | 1.0 |  |  |  |  |
| Good | 1.05 | 0.81 | 1.45 | .37 | .63 |
| Neither | **2.10** | **1.11** | **3.72** | **.01** | **.99** |
| Bad/very bad | **2.65** | **1.02** | **6.68** | **.02** | **.98** |
| Experience of being treated respectfully during last healthcare provider visit, |  |  |  |  |  |
| Very good (ref) | 1.0 |  |  |  |  |
| Good | 0.97 | 0.71 | 1.23 | .59 | .41 |
| Neither | 1.09 | 0.79 | 1.75 | .34 | .66 |
| Bad/very bad | **2.11** | **0.99** | **5.06** | **.04** | **.96** |

CI = confidence interval; Modified-SCIM-SR = modified Spinal Cord Independence Measure (SCIM), self-reported; OR = odds ratio; Ref = reference category.

**Supplemental 3.** General practitioner model outputs

| Variable | OR | 95% CI | | Pr OR <1 | Pr OR >1 |
| --- | --- | --- | --- | --- | --- |
|  |  | **Lower** | **Upper** |  |  |
| Age | 1.02 | 0.92 | 1.15 | .37 | .63 |
| Females (ref is males) | 1.03 | 0.85 | 1.30 | .40 | .60 |
| Receiving daily assistance | 1.01 | 0.82 | 1.28 | .46 | .54 |
| Receiving disability pension | 1.05 | 0.88 | 1.33 | .33 | .67 |
| Education, |  |  |  |  |  |
| Primary/lower secondary (ref) | 1.0 |  |  |  |  |
| Secondary | 1.00 | 0.78 | 1.29 | .50 | .50 |
| Certificate | **1.98** | **1.30** | **2.98** | **.00** | **1** |
| Diploma | **2.47** | **1.45** | **4.24** | **.00** | **1** |
| Bachelor | **2.33** | **1.46** | **3.74** | **.00** | **1** |
| Masters/PhD | **2.84** | **1.51** | **5.46** | **.00** | **1** |
| Geography, |  |  |  |  |  |
| Capital city/metropolitan area (ref) | 1.0 |  |  |  |  |
| Rural | 1.00 | 0.81 | 1.23 | .51 | .49 |
| Remote | 0.95 | 0.72 | 1.17 | .66 | .34 |
| Weekly household income, |  |  |  |  |  |
| <$455 (ref) | 1.0 |  |  |  |  |
| $456 to $909 | 1.01 | 0.82 | 1.25 | .48 | .52 |
| $910 to $1,548 | 1.00 | 0.80 | 1.27 | .49 | .51 |
| $1,549 to $2,969 | 1.15 | 0.90 | 1.69 | .20 | .80 |
| >$2,970 | 1.06 | 0.79 | 1.62 | .38 | .62 |
| Level and completeness, |  |  |  |  |  |
| Incomplete paraplegia (ref) | 1.0 |  |  |  |  |
| Complete paraplegia | 0.94 | 0.71 | 1.15 | .68 | .32 |
| Incomplete tetraplegia | 0.89 | 0.66 | 1.09 | .80 | .20 |
| Complete tetraplegia | 1.09 | 0.81 | 1.74 | .33 | .67 |
| Marital status, |  |  |  |  |  |
| Single (ref) | 1.0 |  |  |  |  |
| Married or cohabitating | 0.95 | 0.73 | 1.14 | .68 | .32 |
| Divorced or widowed | 0.96 | 0.72 | 1.21 | .60 | .40 |
| Unmet healthcare needs | 0.98 | 0.76 | 1.22 | .58 | .42 |
| Modified-SCIM-SR | **0.78** | **0.66** | **0.93** | **1** | **.00** |
| Number of severe secondary conditions | 0.97 | 0.85 | 1.08 | .68 | .32 |
| Injury onset, |  |  |  |  |  |
| Traumatic (ref) | 1.0 |  |  |  |  |
| Non-traumatic | 1.06 | 0.86 | 1.42 | .34 | .66 |
| Time since injury | **1.07** | **0.96** | **1.22** | **.15** | **.85** |

CI = confidence interval; Modified-SCIM-SR = modified Spinal Cord Independence Measure (SCIM), self-reported; OR = odds ratio; Ref = reference category.

**Supplemental 4.** Allied health practitioner use model outputs

| Variable | OR | 95% CI | | Pr OR <1 | Pr OR >1 |
| --- | --- | --- | --- | --- | --- |
|  |  | **Lower** | **Upper** |  |  |
| Age | **0.85** | **0.74** | **0.96** | **1** | **.00** |
| Females (ref is males) | 1.12 | 0.94 | 1.44 | .16 | .84 |
| Receiving daily assistance | **1.96** | **1.46** | **2.63** | **.00** | **1** |
| Receiving disability pension | **1.24** | **0.99** | **1.63** | **.04** | **.96** |
| Education, |  |  |  |  |  |
| Primary/lower secondary (ref) | 1.0 |  |  |  |  |
| Secondary | 0.95 | 0.72 | 1.17 | .66 | .34 |
| Certificate | **1.25** | **0.97** | **1.79** | **.08** | **.92** |
| Diploma | **1.27** | **0.96** | **1.94** | **.09** | **.91** |
| Bachelor | **1.57** | **1.02** | **2.36** | **.01** | **.99** |
| Masters/PhD | **1.39** | **0.97** | **2.30** | **.07** | **.93** |
| Geography, |  |  |  |  |  |
| Capital city/metropolitan area (ref) | 1.0 |  |  |  |  |
| Rural area | **1.15** | **0.96** | **1.51** | **.12** | **.88** |
| Remote areas | 0.99 | 0.80 | 1.19 | .56 | .44 |
| Weekly household income, |  |  |  |  |  |
| <$455 (ref) | 1.0 |  |  |  |  |
| $456 to $909 | 1.07 | 0.91 | 1.39 | .26 | .74 |
| $910 to $1,548 | **1.17** | **0.95** | **1.61** | **.12** | **.88** |
| $1,549 to $2,969 | 0.98 | 0.77 | 1.20 | .57 | .43 |
| >$2,970 | 0.99 | 0.75 | 1.29 | .52 | .48 |
| Level and completeness, |  |  |  |  |  |
| Incomplete paraplegia (ref) | 1.0 |  |  |  |  |
| Complete paraplegia | 1.02 | 0.86 | 1.26 | .40 | .60 |
| Incomplete tetraplegia | 1.00 | 0.84 | 1.18 | .51 | .49 |
| Complete tetraplegia | 0.94 | 0.65 | 1.19 | .65 | .35 |
| Marital status, |  |  |  |  |  |
| Single (ref) | 1.0 |  |  |  |  |
| Married or cohabitating | **1.18** | **0.96** | **1.57** | **.09** | **.91** |
| Divorced or widowed | 1.01 | 0.81 | 1.29 | .48 | .52 |
| Unmet healthcare needs | 0.95 | 0.73 | 1.13 | .69 | .31 |
| Modified-SCIM-SR | **0.86** | **0.72** | **1.01** | **.96** | **.04** |
| Number of severe secondary conditions | 1.02 | 0.94 | 1.14 | .34 | .66 |
| Injury onset, |  |  |  |  |  |
| Traumatic (ref) | 1.0 |  |  |  |  |
| Non-traumatic | 0.93 | 0.70 | 1.11 | .74 | .26 |
| Time since injury | **0.94** | **0.83** | **1.02** | **.89** | **.11** |

CI = confidence interval; Modified-SCIM-SR = modified Spinal Cord Independence Measure (SCIM), self-reported; OR = odds ratio; Ref = reference category.

**Supplemental 5.** Rehabilitation and/or SCI physician model outputs

| Variable | OR | 95% CI | | Pr OR <1 | Pr OR >1 |
| --- | --- | --- | --- | --- | --- |
|  |  | **Lower** | **Upper** |  |  |
| Age | 0.96 | 0.86 | 1.04 | .80 | .20 |
| Females (ref is males) | 0.99 | 0.84 | 1.15 | .55 | .45 |
| Receiving daily assistance | **1.42** | **1.03** | **1.90** | **.01** | **.99** |
| Receiving disability pension | 1.00 | 0.87 | 1.17 | .48 | .52 |
| Education, |  |  |  |  |  |
| Primary/lower secondary (ref) | 1.0 |  |  |  |  |
| Secondary | 0.98 | 0.79 | 1.18 | .57 | .43 |
| Certificate | 1.06 | 0.91 | 1.37 | .29 | .71 |
| Diploma | 1.04 | 0.88 | 1.36 | .36 | .64 |
| Bachelor | 1.05 | 0.89 | 1.35 | .33 | .67 |
| Masters/PhD | **1.57** | **1.00** | **2.44** | **.02** | **.98** |
| Geography, |  |  |  |  |  |
| Capital city/metropolitan area (ref) | 1.0 |  |  |  |  |
| Rural area | **0.79** | **0.59** | **1.01** | **.96** | **.04** |
| Remote areas | **0.87** | **0.62** | **1.05** | **.86** | **.14** |
| Weekly household income, |  |  |  |  |  |
| <$455 (ref) | 1.0 |  |  |  |  |
| $456 to $909 | 0.98 | 0.82 | 1.15 | .58 | .42 |
| $910 to $1,548 | 1.05 | 0.89 | 1.32 | .33 | .67 |
| $1,549 to $2,969 | 1.02 | 0.86 | 1.27 | .42 | .58 |
| >$2,970 | 1.04 | 0.86 | 1.40 | .37 | .63 |
| Level and completeness, |  |  |  |  |  |
| Incomplete paraplegia (ref) | 1.0 |  |  |  |  |
| Complete paraplegia | 1.04 | 0.90 | 1.30 | .33 | .67 |
| Incomplete tetraplegia | 1.00 | 0.84 | 1.16 | .52 | .48 |
| Complete tetraplegia | 1.04 | 0.85 | 1.38 | .39 | .61 |
| Marital status, |  |  |  |  |  |
| Single (ref) | 1.0 |  |  |  |  |
| Married or cohabitating | 1.10 | 0.94 | 1.43 | .18 | .82 |
| Divorced or widowed | 0.88 | 0.62 | 1.07 | .82 | .18 |
| Unmet healthcare needs | 1.07 | 0.91 | 1.37 | .27 | .73 |
| Modified-SCIM-SR | **0.93** | **0.79** | **1.04** | **.86** | **.14** |
| Number of severe secondary conditions | **1.08** | **0.98** | **1.22** | **.08** | **.92** |
| Injury onset, |  |  |  |  |  |
| Traumatic (ref) | 1.0 |  |  |  |  |
| Non-traumatic | 1.09 | 0.93 | 1.45 | .22 | .78 |
| Time since injury | **0.79** | **0.70** | **0.90** | **1** | **.00** |

CI = confidence interval; Modified-SCIM-SR = modified Spinal Cord Independence Measure (SCIM), self-reported; OR = odds ratio; Ref = reference category.

**Supplemental 6.** Medical specialist model outputs

| **Variable** | **OR** | **95% CI** | | **Pr OR <1** | **Pr OR >1** |
| --- | --- | --- | --- | --- | --- |
|  |  | **Lower** | **Upper** |  |  |
| **Age** | **0.94** | **0.84** | **1.03** | **.88** | **.12** |
| Females (ref is males) | 1.00 | 0.85 | 1.17 | .51 | .49 |
| **Receiving daily assistance** | **1.13** | **0.95** | **1.47** | **.14** | **.86** |
| Receiving disability pension | 1.07 | 0.93 | 1.33 | .23 | .77 |
| Education, |  |  |  |  |  |
| Primary/lower secondary (ref) | 1.0 |  |  |  |  |
| Secondary | 0.94 | 0.71 | 1.13 | .69 | .31 |
| **Certificate** | **1.38** | **1.00** | **1.97** | **.03** | **.97** |
| Diploma | 1.15 | 0.93 | 1.70 | .18 | .82 |
| **Bachelor** | **1.86** | **1.26** | **2.68** | **.00** | **1** |
| **Masters/PhD** | **2.08** | **1.27** | **3.25** | **.00** | **1** |
| Geography, |  |  |  |  |  |
| Capital city/metropolitan area (ref) | 1.0 |  |  |  |  |
| Rural area | 0.94 | 0.75 | 1.08 | .75 | .25 |
| Remote areas | 0.97 | 0.79 | 1.15 | .60 | .40 |
| Weekly household income, |  |  |  |  |  |
| <$455 (ref) | 1.0 |  |  |  |  |
| $456 to $909 | 1.03 | 0.89 | 1.26 | .37 | .63 |
| $910 to $1,548 | 0.99 | 0.82 | 1.16 | .55 | .45 |
| $1,549 to $2,969 | 1.01 | 0.84 | 1.23 | .46 | .54 |
| >$2,970 | 1.04 | 0.85 | 1.40 | .37 | .63 |
| Level and completeness, |  |  |  |  |  |
| Incomplete paraplegia (ref) | 1.0 |  |  |  |  |
| Complete paraplegia | 0.94 | 0.74 | 1.09 | .73 | .27 |
| Incomplete tetraplegia | 1.02 | 0.83 | 1.33 | .43 | .57 |
| Complete tetraplegia | 1.06 | 0.92 | 1.32 | .26 | .74 |
| Marital status, |  |  |  |  |  |
| Single (ref) | 1.0 |  |  |  |  |
| Married or cohabitating | 0.99 | 0.84 | 1.16 | .53 | .47 |
| Divorced or widowed | 0.92 | 0.68 | 1.09 | .76 | .24 |
| Unmet healthcare needs | 1.03 | 0.88 | 1.27 | .38 | .62 |
| **Modified-SCIM-SR** | **0.92** | **0.81** | **1.03** | **.90** | **.10** |
| **Number of severe secondary conditions** | **1.11** | **1.00** | **1.26** | **.03** | **.97** |
| Injury onset, |  |  |  |  |  |
| Traumatic (ref) | 1.0 |  |  |  |  |
| Non-traumatic | 1.07 | 0.91 | 1.38 | .28 | .72 |
| Time since injury | 0.98 | 0.89 | 1.06 | .69 | .31 |

CI = confidence interval; Modified-SCIM-SR = modified Spinal Cord Independence Measure (SCIM), self-reported; OR = odds ratio; Ref = reference category.

**Supplemental 7.** Hospitalisations in the past 12-months model output

| **Variable** | **OR** | **95% CI** | | **Pr OR <1** | **Pr OR >1** |
| --- | --- | --- | --- | --- | --- |
|  |  | **Lower** | **Upper** |  |  |
| **Age** | **1.17** | **1.03** | **1.33** | **.01** | **.99** |
| Females (ref is males) | 0.98 | 0.83 | 1.12 | .59 | .41 |
| Receiving daily assistance | 1.06 | 0.92 | 1.34 | .27 | .73 |
| Receiving disability pension | 1.10 | 0.95 | 1.39 | .17 | .83 |
| Education, |  |  |  |  |  |
| Primary/lower secondary (ref) | 1.0 |  |  |  |  |
| Secondary | 0.99 | 0.82 | 1.17 | .54 | .46 |
| Certificate | 1.01 | 0.86 | 1.20 | .46 | .54 |
| Diploma | 0.99 | 0.82 | 1.19 | .52 | .48 |
| Bachelor | 0.98 | 0.80 | 1.14 | .60 | .40 |
| Masters/PhD | 0.96 | 0.72 | 1.13 | .65 | .35 |
| Geography, |  |  |  |  |  |
| Capital city/metropolitan area (ref) | 1.0 |  |  |  |  |
| Rural area | 1.08 | 0.94 | 1.38 | .21 | .79 |
| Remote areas | 1.03 | 0.89 | 1.26 | .39 | .61 |
| Weekly household income, |  |  |  |  |  |
| <$455 (ref) | 1.0 |  |  |  |  |
| $456 to $909 | 0.99 | 0.84 | 1.15 | .53 | .47 |
| $910 to $1,548 | 1.02 | 0.88 | 1.24 | .41 | .59 |
| $1,549 to $2,969 | 0.98 | 0.81 | 1.15 | .57 | .43 |
| >$2,970 | 0.96 | 0.71 | 1.15 | .63 | .37 |
| Level and completeness, |  |  |  |  |  |
| Incomplete paraplegia (ref) | 1.0 |  |  |  |  |
| Complete paraplegia | 1.07 | 0.92 | 1.37 | .26 | .74 |
| **Incomplete tetraplegia** | **0.76** | **0.57** | **1.00** | **.98** | **.02** |
| Complete tetraplegia | 1.03 | 0.84 | 1.36 | .42 | .58 |
| Marital status, |  |  |  |  |  |
| Single (ref) | 1.0 |  |  |  |  |
| Married or cohabitating | 1.01 | 0.87 | 1.19 | .47 | .53 |
| Divorced or widowed | 1.05 | 0.90 | 1.35 | .33 | .67 |
| Unmet healthcare needs | 1.00 | 0.84 | 1.18 | .51 | .49 |
| **Modified-SCIM-SR** | **0.64** | **0.55** | **0.74** | **1** | **.00** |
| **Number of severe secondary conditions** | **1.27** | **1.12** | **1.44** | **.00** | **1** |
| Injury onset, |  |  |  |  |  |
| Traumatic (ref) | 1.0 |  |  |  |  |
| Non-traumatic | 1.02 | 0.88 | 1.25 | .41 | .59 |
| Time since injury | 0.96 | 0.86 | 1.04 | .79 | .21 |

CI = confidence interval; Modified-SCIM-SR = modified Spinal Cord Independence Measure (SCIM), self-reported; OR = odds ratio; Ref = reference category.
